# Academic coaching and decision analysis: Ways of deciding whether to pursue an academic career

**DOI:** 10.1371/journal.pone.0206961

**Published:** 2018-11-15

**Authors:** Ana Sofia Morais, Wasilios Hariskos

**Affiliations:** 1 Department of Research System and Science Dynamics, German Centre for Higher Education Research and Science Studies, Berlin, Germany; 2 Center for Adaptive Behavior and Cognition, Max Planck Institute for Human Development, Berlin, Germany; 3 Chair of Applied Microeconomics, University of Erfurt, Erfurt, Germany; IUMPA - Universitat Politecnica de Valencia, SPAIN

## Abstract

We analyzed and compared the decision-making processes underlying two approaches that academics might use to decide whether to pursue a professorship or an alternative career: academic coaching (a paid service that supports academics with career-related issues) and decision analysis (a method for applying decision theory to real-world decision problems). To this end, we conducted in-depth expert interviews with seven out of 11 academic coaches known to work in Berlin to examine empirically the career decision-making process that they use. Moreover, we demonstrate theoretically how decision analysis can be applied to an academic’s hypothetical career choice problem. A comparison of the two approaches showed that they both advise (i) structuring the decision problem by dividing it into smaller components, (ii) using the academic’s objectives to generate career alternatives, and (iii) quantifying the uncertainty of decision outcomes using subjective probabilities. Moreover, the observed differences in the way the two approaches structure the decision problem suggest ways in which they could inform each other: (i) they could make use of each other’s techniques to help academics define their objectives and generate career alternatives; (ii) academic coaching could, in addition, use decision trees (a hallmark of decision analysis) to represent the structure of the career decision problem, and use simple measurement scales to quantify how much the career options contribute to the academic’s objectives.

## Introduction

Considering the difficulties of becoming a professor, many young academics must deal with the following decision problem: *Should I pursue a professorship or an alternative career*? The problem is particularly relevant to young scientists in Western countries such as Germany, Italy, and the United States, where decreasing budgets and increasing relative costs have led to fewer scientists being offered permanent positions [1]. Taking a closer look at the German case, 29,218 new doctoral degrees were awarded by German universities in 2015 [2]. According to survey results, 20% of these new PhD recipients wanted to become professors [3]. Assuming constant quality across individuals, what are the chances of being appointed to a professorship in Germany? Based on federal statistics, an average of 825 professors can be expected to retire per year in Germany between 2017 and 2024 [2]. In addition, a new German federal program was approved in July 2016 (in German, the *Nachwuchspakt*) that will create 1,000 new positions by 2032. These numbers indicate that, every year, there will be an expected number of 5,844 new PhD graduates who want to become professors, but only about 892 professor positions can be expected to open. That is, every year, there will be about 4,952 new PhD graduates wanting to become professors who will probably not find a professorship in Germany. These individuals can remain in German academia, hired on short-term contracts, with the accumulation of professorship aspirants from one year to the next decreasing the probability that one will be appointed professor. Yet the pursuit of a professorship cannot last indefinitely because of a federal regulation in Germany that limits temporary academic employment to a maximum of 12 years (doctoral education included). Once this limit is reached, researchers can remain in the academic system only if supported by third-party funding.

Recent work has shown that academics may feel discouraged from pursuing an academic career for reasons other than the academic job market [4]. Over time, researchers learn what it means to be a faculty member, primarily through repeated interaction with mentors and peers, and through their own involvement in research and teaching [5–7]. For instance, junior academics may feel discouraged by the difficulty in securing grants to fund their work [8], or by seeing faculty members being detracted from the time they can spend on research due to administrative duties [9].

In this context, junior academics face the decision of whether to pursue a professorship or an alternative career. Past studies suggest that they may experience difficulties in specifying this decision problem, due to limited knowledge about alternative career options [10,11]. The absence of knowledge about career alternatives can be detrimental to career decision making, as one’s career decision can be no better than one’s best alternative. Consistent with the observation that junior academics lack knowledge about career alternatives, survey results have shown that most of them also make a negative evaluation of their nonacademic career prospects in five years’ time [12]. Moreover, the future consequences of one’s decision are uncertain, in that decision makers cannot know for sure what the repercussions of an alternative will be until after deciding [13]. For instance, decision makers cannot know with any degree of certainty if they will eventually find a professorship (or some other career option) should their decision be to pursue one. At the very most, they may try to make a crude subjective assessment of the chances that an alternative will lead to an outcome.

The scope of the present article is to analyze and compare the decision-making processes used in *academic coaching* and *decision analysis*—user-friendly approaches that could aid academics in their decision to pursue a professorship or an alternative career. Academic coaching is the application of coaching to academic careers, whereby a trained coach supports an academic dealing with work- and career-related challenges [14]. We focus on academic coaching for three reasons. First, academic coaching has grown in popularity among academics in Germany [15]. Second, its methods are specifically tailored to academics; third, we aim to make theoretical contributions to the coaching industry in general, which has been criticized for delaying the development of rigorous and coherent theoretical frameworks [16,17]. Decision analysis, in contrast, is based on decision theory, which it seeks apply to real-world decision problems [18]. Decision analysis has been defined as a “prescriptive approach designed for normally intelligent people who want to think hard and systematically about some important real problems” [19]. We focus on decision analysis as a counterpoint to academic coaching for two reasons. First, decision analysis may provide a coherent theoretical framework of decision-making—still absent in the coaching industry [16,17]—that coaches can use to help coachees tackle complex decision problems. Second, we aim to show how a model from the decision-making literature can be applied to academic career choice—a domain where the difficulty often lies in finding alternatives to an academic career, rather than in narrowing down a multitude of options [10,11,20–22]. As we shall see, both academic coaching and decision analysis provide techniques that academics can use to help them identify potential career alternatives.

In this article, we address the following two research questions: First, what do the decision-making processes adopted by academic coaching and decision analysis look like? Examples of process-related topics include how career alternatives are generated and evaluated, or how uncertainties are assessed. Second, to what extent could academic coaching and decision analysis inform each other, with respect to how they guide the career decision-making process of academics? To answer these questions, we analyze new interview data that we gathered from seven out of 11 credentialed academic coaching professionals that are known to work in Berlin. Our analysis of the qualitative data focuses on one aspect of the coaching intervention, that is, the decision-making approach that coaches use to guide academics throughout the process of figuring out their career preferences. Moreover, we provide a new theoretical demonstration of how decision analysis can be applied to career choice problems like the one academics typically face—problems with few known career alternatives and little knowledge about them. Whereas our application of decision analysis is theory-driven, our analysis of the decision processes used by academic coaches is empirically-based, grounded in the coaches’ personal, context-specific knowledge that they acquired through experience. By analyzing and comparing the decision-making processes adopted by each approach, the present work helps build a bridge between two seemingly unrelated approaches.

## Academic coaching

Although definitions of professional coaching abound, there is general agreement that coaching is a goal-directed process designed to help mentally healthy individuals control and direct their resources to create purposeful and positive changes in their personal or professional lives [23]. The notion of the coach as advice giver is somewhat controversial, with many coaches emphasizing a nondirective approach that seeks to help coachees find their own solutions [24]. This sets coaching apart from other helping relationships such as counseling or consulting, where expert knowledge tends to be conveyed in the form of diagnoses or advice. Academic coaching emerged in Germany in the mid-2000s under the term *Wissenschaftscoaching*, as an application of professional coaching to academic careers [14].

We conducted in-depth interviews with professional academic coaches to gain insight into the decision-making process through which they guide academics to help them decide between a professorship and one or more alternative careers. Academic coaches support academics dealing with the challenges of the different stages of an academic career [14], including (i) making career decisions, (ii) developing time management and task prioritization skills, and (iii) developing the leadership skills of newly appointed professors [25]. It is recommended that academic coaches have professional coaching qualifications, along with detailed knowledge about the academic system that they acquired firsthand, for example, as doctoral students or science managers [14]. Academic coaches working in Germany are either self-employed or employed by a consulting company or university. The coaching typically unfolds over multiple sessions, at an average cost of 100 to 160 euros per hour [26], with most academics paying for the coaching out of their own pockets.

### Methods

#### Interview guideline

We developed the interview guideline with the goal of answering the following research question: How do academic coaches guide their clients throughout the process of deciding between a professorship and one or more alternative careers? The interviews were semi-structured and provided coaches with the opportunity to describe their own views and coaching approach. Coaches were asked four classes of questions concerning (i) what they did as academic coaches, (ii) who their coachees were in terms of their background and professional experience, (iii) what reasons led coachees to seek coaching support, (iv) how coaches helped their clients throughout the process of deciding between alternative careers, in addition to some optional concluding questions. We deliberately framed questions (iii) and (iv) without inducing the specific decision of whether to pursue a professorship or an alternative career; this allowed the topic to emerge naturally and helped us access its relevance for academic coaching practice. Two versions of the interview script, in English and German, are provided in S1 Appendix.

#### Identification and selection of cases

We searched online for the homepages of academic coaches working in Berlin. The online search returned results for ten coaches, whom we invited to take part in the study. Six agreed to be interviewed. At the end of sixth interview, we asked the coach to name other Berlin-based academic coaches to check whether we may have had left other potential cases unidentified. The sixth coach named most of the coaches that had already been invited to the study, in addition to one new coach, whom we also interviewed afterwards. Hence, we interviewed a total of seven coaches, six found via online search, and one reached through referral. The analysis of the interview data only began once all interviews had been conducted.

Qualitative researchers often keep conducting interviews until they have reached “saturation”—when the collection of new data does not shed any further light on the issue under investigation [27]. Whether or not we have reached saturation of possible responses with seven interviews is a moot point. Yet a sample of seven interviews can be considered sufficient to uncover themes and patterns if they are expected to be highly prevalent among a small, elite group of specialized academic coaching experts [28]. Our interviews have provided us with high-quality pieces of information about academic coaching practices, offering a rich, complex, and detailed account of how the interviewees guide their clients throughout the career decision-making process.

#### Characteristics of the cases

The seven academic coaches who took part in the study (six women and one man) held a PhD degree from a German university, in disciplines such as biology, physics, education, and German studies. Additionally, they were certified by an accredited coach-training organization. The interviewees differed in how much coaching experience they had, ranging from 4 to 17 years. Five coaches were self-employed, one was employed by a university, and one was employed by a university and self-employed at the same time. In addition to offering individual coaching services, most self-employed coaches work as trainers, giving group workshops at higher education institutions in Germany. These workshops cover a variety of topics such as career development, scientific presentation, and time management. Coaches employed by universities typically work in the administrative office of a graduate research school, where they offer individual coaching services to PhD candidates and postdocs affiliated with the school. Note that our interviews focused on individual coaching rather than on group coaching workshops because it is usually in the former context that coaches provide career decision-making support to academics.

#### Interview sessions

Five interviews were conducted in German and two in English, with the same questions used as a guideline for each interview. The interviews lasted approximately 1 hr 15 min. Four interviews took place in May 2016, and the other three in June 2016. The conversations were tape recorded with the oral consent of the interviewees and subsequently transcribed. In total, the seven transcripts have 104 pages, with single spacing between lines and font size 11.

#### Qualitative data analysis

We analyzed the data by applying thematic analysis, as described by Braun and Clarke [29]. Thematic analysis is a method for identifying, analyzing, and reporting themes or patterns in qualitative data. The goal of our analysis was not to describe the whole data set, including, for instance, all the reasons why academics seek coaching support, but rather to provide a detailed account of one aspect―the decision-making process through which coaches guide academics to help them decide between a professorship and one or more alternative careers. Therefore, although a coaching relationship has “the potential to be a major force for the promotion of wellbeing and performance enhancement for the individual”[16, p. 20], our qualitative analysis focuses on one aspect of the coaching intervention, namely, the career decision-making process applied by the coach.

We began by identifying and coding the features of the data that relate to the career decision-making process. A code has been defined as “the most basic segment, or element, of the raw data or information that can be assessed in a meaningful way regarding the phenomenon” [30, p. 63]. The relevant data extracts were coded by means of an inductive, data-driven approach, which sidesteps the use of a preexisting coding scheme. In a subsequent phase of the analysis, the different codes were combined to form overarching themes. We identified these themes at the semantic level, without looking deeper for underlying assumptions and ideologies in a psychoanalytic fashion. Next, we refined and revised the set of themes to make sure that data within themes fit together meaningfully, while at the same time the different themes could be clearly distinguished from each other. We also identified whether a theme contained any subthemes that could be used to give it a more organized structure.

#### Ethics statement

The interviews and the subsequent data analysis were conducted according to the principles of good scientific practice of the German Centre for Higher Education Research and Science Studies. The board of directors of the Department of Research System and Science Dynamics, which also assumes the function of an institutional review board, evaluated and approved the study. All interviewees provided informed consent via e-mail before the interview was scheduled, and again orally at the beginning of the interview session.

### Results

When coaches were asked about how they provided decision-making support to academics, they generally indicated seeing their role as nondirective and facilitative, as opposed to the advice-giving role of a supervisor. This means that coaches did not instruct coachees to choose any course of action:

*You just do this thing of taking the blinds off their eyes and they see their own solution*.

*I usually do not say: “I think it would be good for you to become this and that.” […] Usually you do not give advice as a coach*. *It is more about making clear for the clients what is going on in their heads*.

*Coaching means help for self-help. […] People don’t come to me and say: “[…] what is the best way?” and then I tell them, “Yes, do this.” This is not the case*. *That’s more, for example, the case of a supervisor… He gives advice. (Authors’ translation)*

To improve their clients’ ability to make career choices, the coaches prompted them to structure the decision problem by breaking it down into smaller components. Three types of components were identified in the interview data: (i) a small consideration set of career alternatives, (ii) the consequences of choosing an alternative, and (iii) the uncertainties that influence the outcome of choosing an alternative. Separating a problem into its constituent parts, one coach argued, makes the problem easier to tackle and reduces the fear of making bad decisions. The coaches further indicated that they applied visualization techniques to support the development of the decision-problem structure. The resulting representations, however, did not seem to have a fixed type of structure, such as a mind map, flowchart, or inheritance hierarchy:

*If it is some sort of big problem, then it’s all about dividing it into smaller aspects. This reduces fear and is easier to tackle. […] Usually, I write things down while talking to people*. *Here on a flipchart, for instance, like notes, key points, and this often helps a lot. (Authors’ translation)*

*Most often the point is […] to clarify one’s own motives as a basis for decision making. This is often like porridge, a gray mass. […] In most cases, the point is to visualize this gray soup that is in one’s head and also in one’s feelings […]. For instance*, *I work a lot with Post-it notes on the pin board, with different colors, with symbols. (Authors’ translation)*

#### Consideration set of career alternatives

The consideration set of career alternatives―composed by two or more alternatives, one of them being to pursue a professorship―was mentioned spontaneously by all coaches as one of the primary components of the decision problem. After the coach referred to career alternatives, the interviewer explicitly probed him or her to describe possible strategies for identifying alternatives. The coaches mentioned three elicitation strategies that are helpful to identify potential career alternatives. All the strategies involve finding key pieces of information―about career objectives, role models, and favorite tasks―that coach and coachee can use as cues or stimuli for generating appropriate career options.

Strategy 1 uses the objectives of the coachee as cues for generating potential career alternatives. The strategy distinguishes between fundamental objectives and means objectives: Whereas a fundamental objective represents something essential that the decision maker really wants to accomplish, a mean objective represents a way station in the progress toward a fundamental objective. The strategy involves (i) thinking about the ideal, long-term future in order to identify one’s fundamental objectives, (ii) identifying the means objectives that one wants to achieve in order to reach those fundamental objectives, and finally (iii) asking “how” one could achieve the means objectives believed to bring one closer to one’s fundamental, long-term goals.

*I invite them to forget about reality, and how hard reality is, and think what they would want if everything were perfect*, *so that you can find the “what.” And then we can talk about the “how.”*

*Then I say: “It’s your 50th birthday and someone delivers an honorific speech. What would you be proud of?” And then the person says […]: “In 10 years […] I earned money […]”, or “I am now at a foundation and I am the manager of a department.” Then, I say: “Okay, let’s come back to today, right? From the 50th to the 40th year of your life.” And I say: “What does this mean to you on the level of objectives? Is it an objective for you to have a management position? Is it an objective for you to earn 50,000 euros, […]*, *100,000 euros […]?” (Authors’ translation)*

Strategy 2 is to search for role models. A role model has been defined as a “cognitive construction based on the attributes of people in social roles an individual perceives to be similar to him or herself to some extent and desires to increase perceived similarity by emulating those attributes” [31, p. 136]. An individual may also have negative role models, who illustrate behaviors that the individual desires to avoid. One coach explained that searching for positive and negative role models can help academics learn about career options that they may or may not have imagined for themselves. Moreover, the coach prompted coachees to identify the key attributes that make a role model positive or negative:

*One strategy is to search for role models, namely, positive and negative ones. Often, they come and say: “I have a superior; she is a professor, and when I see how she works, by no means do I want to be like her*. *I want to leave academia.” Then I say, for example: “Well, then you have a wonderful role model there. […] What is it that you see in this example […] that you by no means want?” Often people answer: “I don’t want to work day and night. […] I don’t want to remain without children.” And then I say: […] “What’s your counter role model? Whom have you met, or will you meet in the next weeks about whom you think: “That’s interesting, what he or she is doing.” […] What is it that you find exciting? What is it that the person has that I would like to have as well?” (Authors’ translation)*

Strategy 3 uses the set of tasks or activities that the coachee enjoys and excels at doing as a cue to search for jobs where those tasks play a role:

*I ask: “What do you enjoy doing?” […] And then someone says*: *“Actually, I like teaching.” […] Then, if I would ask, for example: “Well, how about transferring what you like to do at the university to a job in the free market economy? So, where in this field could you, for example, enjoy working?” (Authors’ translation)*

*For instance, I think it’s very important to give some sort of impetus so that they find out what they really enjoy doing. I ask, for example, whether they have experienced a sense of achievement during the last year; something they really enjoyed doing at work, or maybe in their private life; and I try to let them talk about it*, *things they can do well or where they experience some success. (Authors’ translation)*

Overall, the three elicitation strategies probe individuals to generate key pieces of information―career objectives, role models, and favorite tasks―that can be used as queries for searching their minds and the external world (e.g., the Internet, their social circle) for potential career alternatives.

#### Future consequences and uncertainties

We now turn to the second and third components of the decision problem: the consequences that may occur after an alternative is chosen, and the uncertainties influencing what those consequences will be. Coaches indicated that they seek to improve the coachees’ awareness of the possible consequences of choosing an alternative and the associated feelings by asking them to anticipate their state of mind if they had decided to pursue a particular career:

*The person should imagine that he or she had made a decision. […] Then, I say: “[…] how do you feel about it now?” So, we pretend the person has made the decision*. *The point of this exercise is to confront the person with the consequences of the decision. (Authors’ translation)*

*“If a vacancy opens at [a research organization in Berlin], would you be willing to move here with your whole family? […]” This is to make clear how many consequences follow a specific decision*. *Or I say: “Okay, you are a professor now. […] What would your inaugural lecture be like? With what topic would you start? How do you stand there? Do I feel the enthusiasm? Do I feel the joy of being a professor now?” (Authors’ translation)*

Moreover, coaches seek to increase academics’ awareness of the uncertainties influencing the outcomes of deciding to pursue a professorship, and they prompt academics to quantify their belief about the likelihood of being appointed professor with a subjective probability:

*More than 90% of PhD graduates leave academia. To many this is not clear*. *It is like a funnel and it is really, really hard to go all the way to the end. (Authors’ translation)*

*And then you discuss. […] For example: “How many professorships are there in Germany, in a certain field?” Then, “There are two or so*.*” Then, “How high are the chances?” (Authors’ translation)*

The subjective probability of getting a job is influenced both by the person’s knowledge about the job market’s supply and demand (gleaned, for instance, from job market data), and by the self-perception of the person’s quality as an applicant.

Although coaches may mention what they believe the coachee’s chances are of finding a professor position, mostly they entrust other academics from the same discipline with the task of helping the coachee assess his or her individual chance of being appointed professor. The reason, the coaches argued, is that they lack the discipline-specific knowledge that is necessary to judge the strength of an academic’s profile when applying for professorships in any given discipline:

*Well, I would never say “You have no chance”; rather, [I might say:] “In comparison to colleagues who are maybe 5 years younger, who don’t have children, who have good funding, who published more, who did [their] dissertation in a different context, the probability that you get there is rather low.” I confront them with this*, *but I also tell them: “You know, I can’t and I don’t want to discourage you from doing it. Talk to people […], go to your discipline, show your profile and ask how high the chance is that you will get there.” (Authors’ translation)*

As these quotes illustrate, the discourse of the coaches about future consequences and uncertainties focused mainly on the pursuit of a career as a professor. Although choosing a nonacademic career often entails uncertain outcomes, we found no evidence that the coaches prompted their clients to specify such outcomes and their respective chances of occurring.

In the next section, we draw insights from decision analysis, an alternative decision support method that academics may use to help them decide between a professorship and one or more career alternatives. While academic coaching is a helping relationship, decision analysis can be applied by decision makers alone, once they have learned the necessary concepts and techniques. In the last section, we examine the similarities and differences between academic coaching and decision analysis.

## Decision analysis

Decision analysis emerged in the early 1960s with the application of decision theory to real-world, complex problems [18]. It is aimed at helping the average person make difficult and important decisions that can, but need not be, career-related. The approach aims not to identify an alternative that must be blindly chosen but to transform “opaque decision problems into transparent decision problems by a sequence of transparent steps”[32, p. 680]. Decision analysis is best thought of as an “information source” [33] that can already be considered valuable “if the decision maker has actually learned something about the problem and his or her own decision-making attitude”[34, p. 8]. When the repercussions of the decision are not fully known, decision analysis can increase the chances of, but not ensure, positive outcomes.

### The process of decision analysis

The general features of decision analysis can be categorized in four steps [33,35–37]. Step 1 identifies the decision to be made and the relevant objectives that the decision maker wants to achieve. As for most important choices, the decision of what career to pursue is typically based on multiple objectives. Decision analysis makes the distinction between fundamental objectives (e.g., buying a house) and means objectives (e.g., a higher salary). According to this approach, asking “Why is objective *X* important?” can help the decision maker find out if *X* is important in itself, or if it is important because it helps achieve another objective *Y*.

Step 2 specifies a set of two or more alternatives that may allow the decision maker to achieve his or her objectives. In decision analysis, the search for good alternatives is driven by the decision maker’s objectives. Alternatives can be generated by asking “How can I achieve objective *X*?” for each individual objective, both means objectives and fundamental objectives. Whereas asking “Why?” in Step 1 allows the decision maker to identify fundamental objectives based on means objectives, asking “How?” brings the decision maker back to means, leading him or her toward alternatives that can be understood as the ultimate means [13].

Step 3 determines the possible consequences that an alternative may have for the decision maker’s fundamental objectives. Since the repercussions of a career choice are often not fully known, a subjective probability distribution must be specified that indicates the decision maker’s belief about the likelihood that a chance outcome will occur. How alternatives are evaluated depends on the nature of the objectives. While an objective such as “good financial status” can be measured in terms of money, and “be a present parent” can be measured in hours, objectives without a natural scale (e.g., “good corporate culture”) require a customized rating scale.

While Steps 1–3 are mostly qualitative, Step 4 relies on quantitative analyses to identify the decision maker’s preferred alternative. The preferences of the decision maker are modeled with a multiobjective utility function that reflects the risk attitude of the decision maker regarding each objective and his or her willingness to make trade-offs between conflicting objectives [33]. The estimation of the free parameters of the utility function requires empirical data about the decision maker’s behavior in other risky, multi-attribute choice situations, which can be gathered using different experimental procedures. When the decision maker lacks information about the probabilities with which different chance outcomes may occur, these also must be estimated from data (e.g., job market data). These computations can become very complex as the number of objectives and alternatives under consideration increases. For that matter, it has long been debated in the decision sciences whether the average decision maker is able to perform these complex procedures, and whether simpler processes can lead to equally good or even better decisions [38].

Although the quantitative analyses performed in Step 4 were long thought to be the most crucial stage of a decision analysis, they are now believed to be secondary relative to the qualitative decision structuring that takes place in Steps 1–3. In the words of Keeney [37]:

“I used to think that quantitative aspects were the most important parts of any decision analysis. Now I believe that the qualitative parts are the most important. If you do not have the right problem, objectives, alternatives, list of uncertainties, and measures to indicate the degree to which the objectives are achieved, almost any [quantitative] analysis will be worthless. No quantitative analysis has ever been done that did not rest on a foundation of qualitative structuring. Furthermore, by being clear about the qualitative aspects, one can resolve many decision problems without an analysis. If your objectives are made crystal clear, the best alternatives may be obvious. If you create an alternative that is terrific, just choose it and that decision problem is over.” (p. 200)

In the following, we show theoretically how decision analysis can be applied to an academic’s career choice problem, where a career as a professor is compared with one or more alternative career options. Specifically, we demonstrate how a graphical representation called a decision tree can be used by academics to structure the information compiled during the qualitative stages of decision analysis.

### Using a decision tree to structure an academic’s career decision

**Fig 1:**
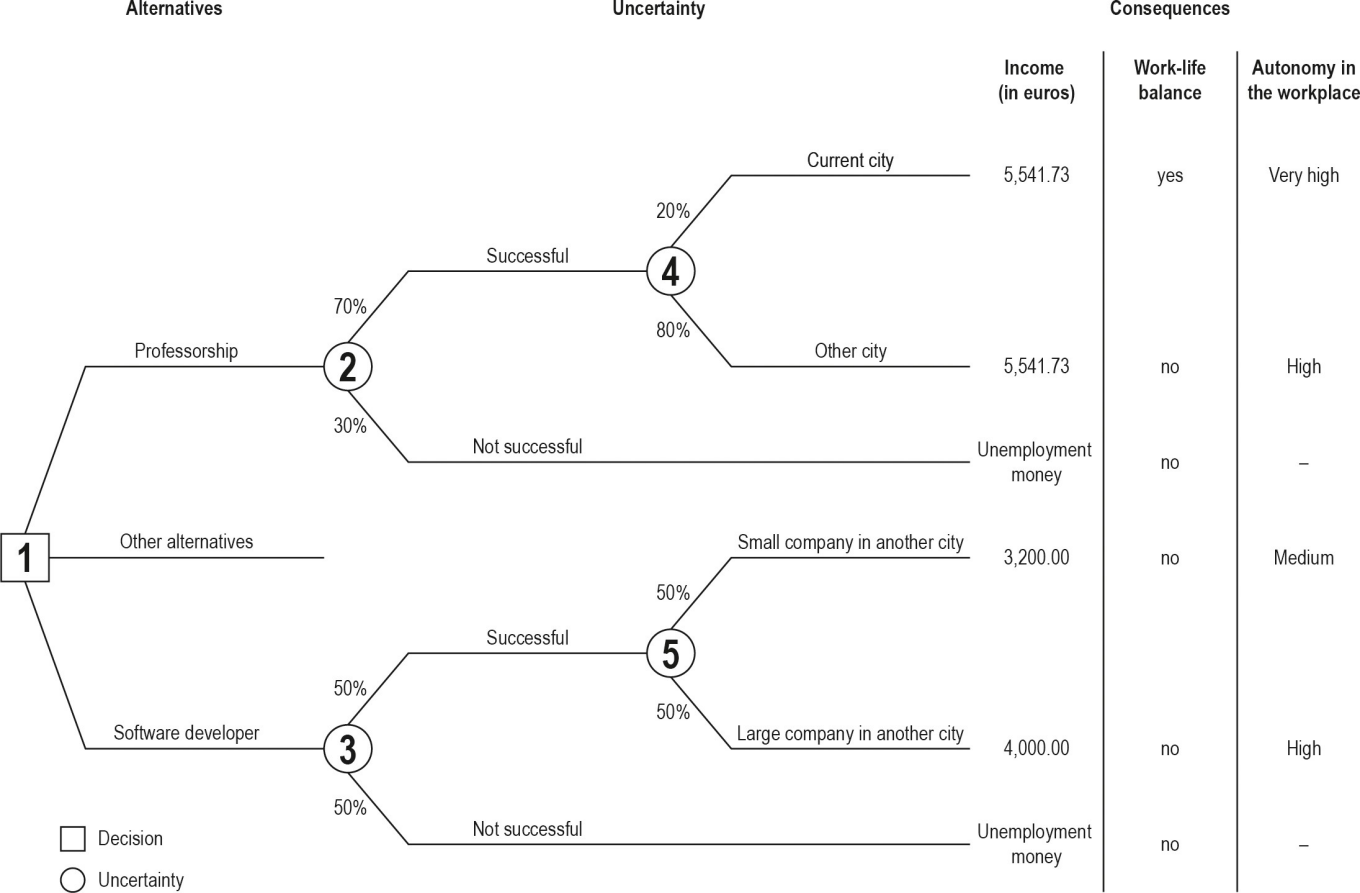
Example of a decision tree. For any given chance outcome, the decision maker (square labeled 1) assigns a subjective probability between 0% and 100%; for any given chance event (circles labeled 2–5), the probabilities of its outcomes must add up to 100%. Income is measured objectively by gross monthly income [39,40]. Work–life balance is evaluated subjectively by the decision maker according to whether current private life arrangements can be maintained. Autonomy in the workplace is evaluated subjectively by the decision maker using a rating scale (very low, low, medium, high, very high); the hyphen indicates that autonomy in the workplace cannot be evaluated if the decision maker is not successful in finding a job.

A decision tree is a useful tool for depicting the architecture of a decision problem—namely, the interrelationships among alternatives, the uncertainties involved, and the consequences that may follow—as perceived by the decision maker. Fig 1 shows a decision tree depicting a hypothetical career decision problem of an academic. Our aim in presenting and analyzing this tree is not to show what the career decision problem of the average academic looks like but rather to illustrate how decision trees can be applied to career decision problems in academia.

The decision tree begins at the point where the decision maker decides, with the initial branches (upper and lower) representing two career alternatives: pursuing a professorship or becoming a software developer in the private sector. The middle branch indicates that it is possible to specify more than two alternatives in a decision tree. The professorship and software developer alternatives each lead to a chance event. The outcome of each chance event can be either “successfully getting a position” or “not getting a position.” The decision maker believes that the success probability of professorship is slightly higher than the success probability of software developer, as indicated by the subjective probabilities attached to the outcome branches. Since the probabilities are inherently subjective, two decision makers comparing the same alternatives may attach different probabilities to the same outcomes.

Subjective probabilities can be assigned to events by relying on one’s own knowledge about the job market’s supply and demand, or one’s self-perception of one’s quality as an applicant. Additionally, the decision maker can increase the accuracy of his or her probability judgements by consulting existing information sources (e.g., job market statistics, research articles), or asking experts (e.g., senior faculty members, academic coaches). Lastly, if a subjective probability cannot be ascertained with pinpoint precision, the decision maker can define the range in which the probability may fall and take the central value [13]. For example, if the success probability of professorship falls between 50% and 90%, the decision maker can compare the alternatives using 70%.

The professorship alternative leads to three terminal outcomes: (i) being appointed to a professor position in the city where the decision maker lives at present with probability 70% ∙ 20% = 14%, (ii) being appointed to a professor position in another city with probability 70% ∙ 80% = 56%, and (iii) not getting a professor position with probability 30%. Hence, conditional on being successfully appointed to a professor position, the probability that the position will be in another city is four times higher than the probability that it will be in the city where the decision maker lives at present.

Concerning the software developer alternative, the decision maker believes that he or she could only find work in another city. The alternative leads to three terminal outcomes: (iv) getting a job at a small company with probability 50% ∙ 50% = 25%, (v) getting a job at a large company with probability 50% ∙ 50% = 25%, and (vi) not getting a job with probability 50%. Hence, conditional on being successful in getting a job as a software developer, the probability that the position will be at a small company is the same as the probability that it will be at a large company. Note also that different alternatives can be affected by different kinds of uncertainty: Whereas the uncertainty affecting the professorship alternative is about location, the uncertainty affecting the software developer alternative is about the size of the company.

Each of the six terminal outcomes differs in terms of how well it fulfills the three objectives of the decision maker, each corresponding to some desired job characteristic: (i) income, (ii) work–life balance, and (iii) autonomy in the workplace (i.e., control over how the workday is organized and the pace at which the employee works). According to the 2017 World Happiness Report [41], these three objectives are strong predictors of various measures of happiness. The consequences of each terminal outcome are summarized, by objective, at the tips of the tree. In keeping with the World Happiness Report, we assume that (i) a higher income is preferred to a lower income, (ii) a good work–life balance is preferred to a poor work–life balance, and (iii) higher individual autonomy in the workplace is preferred to lower autonomy.

This decision tree illustrates how structuring a decision problem can reveal the best alternative and render complex, quantitative analyses unnecessary. It turns out that the professorship alternative has advantages, and no disadvantage, relative to the software developer alternative and can thus be considered the best alternative for the decision maker based on the current problem specification. First, the event of not getting a professor position is less likely than the event of not getting a software developer position, while having no further disadvantages for the decision maker’s objectives. Second, conditional on being successful, the salary of a professor (not influenced by location) is higher than the maximum salary of a software developer (achieved at a large company). The consequences of becoming a professor for the decision maker’s work–life balance and individual autonomy are equally good or even better than those of becoming a software developer. Hence, the decision maker could decide immediately to pursue a professorship, while sidestepping complex quantitative analyses.

Sometimes, however, it is not possible to resolve a decision problem simply by representing its structure. This is the case when the alternative with better consequences for the decision maker’s objectives entails a higher risk, or when the alternatives have disadvantages on different objectives. In such situations, the decision maker may try to identify the alternative with the highest expected utility by executing the quantitative operations outlined in Step 4. Alternatively, the decision maker could simply revise the decision tree, as the decision could turn out to be obvious with an improved representation. Possible revisions include (i) revising the objectives or adding other important missing objectives that could make an alternative clearly more attractive, (ii) generating better alternatives based on the new set of objectives, and (iii) trying to make an existing alternative more attractive by improving the subjective probability of the chance outcomes. For instance, the decision maker could ask senior scholars for advice about how to increase the probability of becoming a professor.

## A comparison of academic coaching and decision analysis

The preceding sections described the decision-making processes of academic coaching and decision analysis, applied to the situation of helping an academic decide between pursuing a professorship or one or more alternative careers. In the present section, we identify the shared features and key differences in how academic coaching and decision analysis guide the career decision-making process of academics. In addition, we examine to what extent the two approaches could inform each other concerning the decision processes that they use. Our comparison of the two approaches should, however, be read with a caveat in mind—our knowledge of academic coaching is based on complex, rich and detailed qualitative accounts, but it cannot be guaranteed that the seven coaches we interviewed are representative of all individuals engaged in academic coaching as a profession.

### Similarities and differences

There are three major similarities between the two approaches. First, they both adopt a “divide and conquer” approach to decision making that breaks down the decision problem into smaller components to identify the best alternative. The problem’s constituent components are the same in the two approaches, namely, objectives (means and fundamental), alternatives, consequences, and uncertainties. Second, both approaches recommend generating alternatives by asking how one could achieve the means objectives believed to bring one closer to one’s fundamental, long-term goals. Finally, both approaches use subjective probabilities to quantify the chances that choosing an alternative will lead to different possible outcomes.

Although both approaches aim to develop a composite structure of the decision problem, they use different techniques to achieve this goal. Table 1 lists the decision-structuring techniques used by the two approaches at different stages of the decision process.

**Table 1:** Decision-structuring techniques used by academic coaching and decision analysis.

Stage of the decision process	Academic coaching	Decision analysis
Visually represent the decision situation	Write ideas down freely, without inserting them into a fixed type of structure.	Use decision tree to represent interrelationships among alternatives, uncertainties, and future consequences.
Identify fundamental objectives	Characterize ideal future to determine one’s fundamental objectives.	Ask “Why is objective *X* important” to go from means objectives to fundamental objectives.
Generate alternatives	Use means and fundamental objectives, role models, and favorite tasks as cues to generate alternatives.	Ask “How can objective *X* be achieved?” for each objective, both means and fundamental.
Evaluate alternatives	Simulate postdecision state of mind to evoke potential consequences of the decision and associated feelings. Focus on the professorship alternative.	Use measurement scales to quantify how much an alternative contributes to each fundamental objective. Evaluate all alternatives.
Assess uncertainties	Focus on the uncertainty of being appointed professor.	Consider uncertainties that may affect each of the alternatives.

The first stage concerns the representation of the decision-problem structure. The representation produced in academic coaching is typically a freely generated collection of ideas that is not organized into a defined, conventional type of structure for representing information. Decision analysis, in contrast, uses a decision tree to represent the interrelationships among alternatives, their uncertainties, and future consequences.

The second stage concerns how fundamental objectives are identified. Academic coaches prompt coachees to think about their ideal, long-term future to identify fundamental objectives. Decision analysis, in contrast, asks “Why is objective *X* important?” in an iterative fashion, to find out whether an objective is fundamentally important or just a means to achieve another objective.

The third difference between the two approaches is about the way alternatives are generated. As mentioned above, both approaches generate alternatives based on fundamental and means objectives. Academic coaches, however, also apply additional elicitation strategies that use information about the client’s role models (positive and negative) and favorite tasks as stimuli for generating alternative career options.

The fourth stage of the decision process refers to how alternatives are evaluated. To anticipate the potential consequences of an alternative and associated feelings, academic coaches ask their clients to imagine that they had chosen an alternative and to simulate their state of mind in that situation. In decision analysis, in contrast, alternatives are systematically evaluated in relation to each fundamental objective; using measurement scales, the decision maker quantifies the extent to which each alternative contributes to each fundamental objective. It follows that the objectives of the decision maker occupy a more central role in decision analysis than in academic coaching: Whereas both approaches use objectives to generate alternatives, only decision analysis considers objectives when determining the consequences of alternatives. Finally, whereas academic coaches mainly evaluate the future consequences of deciding to pursue a professorship, decision analysis evaluates the consequences of all alternatives under consideration.

The last difference between academic coaching and decision analysis concerns how uncertainties are dealt with. As for the analysis of a decision’s future consequences, the analysis of uncertainty in academic coaching focuses mainly on the decision to pursue a career as a professor, although presumably nonacademic career alternatives are subject to uncertainty as well. In decision analysis, the decision maker systematically considers the uncertainties that may affect each of the alternatives in the consideration set.

### To what extent could academic coaching and decision analysis inform each other?

Now that we have compared the career decision-making processes applied by academic coaching and decision analysis and found important differences between the two approaches, let us consider the question of how they could inform each other. As mentioned earlier, the reader should keep in mind that our knowledge of academic coaching was gleaned from in-depth interviews with seven academic coaches who cannot be guaranteed to be representative of all individuals engaged in academic coaching as a profession. We begin by considering how could decision analysis inform academic coaching with respect to one aspect of the coaching intervention, namely, the career decision-making process that coaches apply to provide decision making support to their clients.

The need to consider potential consequences and uncertainties was mostly mentioned in the interviews in relation to a career as professor, with less emphasis being given to the consequences and uncertainties of non-academic career options. Yet positions outside of academia are rarely guaranteed to academics, and the consequences of choosing to pursue a nonacademic career can often not be known beforehand. By focusing mostly on the uncertainties of the academic career, coaches may implicitly convey the impression, not necessarily accurate, that nonacademic career alternatives involve less risk than pursuing a career as university professor. This could be avoided by using a decision tree to represent the interrelationships between the different components of the career choice problem, enabling coach and coachee to consider all uncertainties involved.

In addition, academic coaching could make use of the techniques proposed by decision analysis to help decision makers identify their fundamental objectives and generate career alternatives. Specifically, the strategy of asking why a certain objective is important (to find one’s fundamental objectives) and how each objective could be achieved (to find alternatives) could provide an interesting complement to the coaching toolbox of decision structuring techniques.

Finally, academic coaching could use simple measurement scales (like the ones depicted in Fig 1) to quantify how much each career option contributes to each of the coachee’s fundamental objectives. Our interview data suggests that academic coaches resort mainly to a simulation of the postdecision state of mind to evoke potential consequences of the decision and associated feelings. This holistic assessment of the consequences of choosing a career alternative could be complemented by the more systematic approach used by decision analysis, that involves assessing the consequences of an alternative at the level of each objective.

And how about academic coaching, how could it inform decision analysis with respect to advising academics on how to make career choices? The techniques of academic coaching can help academics specify objectives and generate career alternatives during the decision structuring process, which decision analysts believe to be the most important part of a decision analysis [37]. While decision analysis is taken to be a domain-general technique for solving preferential choice problems, its application to academic career choice could be strengthened by using domain-specific techniques that academic coaches have developed through experience. Examples of such techniques are to imagine one’s ideal future to find fundamental objectives, and to use one’s favorite tasks and role models (positive and negative) to facilitate the generation of career alternatives. Although these techniques are less systematic than the techniques proposed by decision analysis, they can help academics specify the qualitative components of the decision tree.

## Discussion

This article was aimed at analyzing and comparing the decision-making processes used in academic coaching and decision analysis—two approaches that academics could use to aid their decision to pursue a professorship or an alternative career. To this end, we provided new interview data from seven academic coaches to characterize the decision-making process that they use to support their clients, at an average cost of approximately 100 to 160 euros per hour. Moreover, we gave a brief overview of decision analysis and provided a new theoretical demonstration of how this approach can be applied to an academic’s career choice problem. Finally, we compared the decision processes applied in academic coaching and decision analysis to build a bridge between the two approaches and find ways in which they could inform each other with respect to the career decision-making processes that they use.

Our results showed that the two approaches advise (i) structuring the decision problem by dividing it into smaller components (e.g., alternative career options and the uncertainties influencing them), (ii) using objectives to generate career alternatives, and (iii) quantifying the uncertainty of decision outcomes using subjective probabilities, but their approaches to structuring the decision problem are not the same. For instance, whereas decision analysis uses a decision tree to graphically represent the decision problem, academic coaching relies on no fixed data structure. Furthermore, the objectives of the decision maker are more central to decision analysis than to coaching: Whereas both approaches use objectives to generate alternatives, only decision analysis considers objectives when specifying the consequences of alternatives.

Based on the observed differences, we identified possible ways in which academic coaching and decision analysis could inform each other. First, the two approaches could make use of each other’s techniques to find fundamental objectives and facilitate the generation of career alternatives. Moreover, academic coaching could benefit from (i) constructing a decision tree to represent the career decision problem and (ii) using simple measurement scales to quantify how much the career alternatives contribute to each of the coachee’s fundamental objectives.

Overall, these results suggest that academic coaching could take more insights from decision analysis than decision analysis could take from academic coaching. To some extent, this can be explained by the fact that decision analysis was designed to help people solve decision problems. Academic coaching, on the contrary, was not only developed to help academics decide between alternative career options, but rather to help them deal with the challenges of the academic career (be them decision-making problems or not), and in that way enhance their performance and well-being.

One limitation of the present work is that our knowledge of academic coaching was gleaned from in-depth interviews with only seven academic coaches, all based in Berlin. This small sample cannot be guaranteed to be representative of all professional academic coaches working in Germany, let alone in other countries with different academic systems. So, although our interviews yielded high-quality pieces of information about academic coaching practices, the lack of representativeness of our sample does not allow us to generalize our findings to all individuals who are engaged in academic coaching as a profession. A second limitation of our work is that the self-reported data is not direct evidence for what academic coaches do, but only for what they told us that they do. Future work could address this limitation by observing the academic coaching sessions directly instead of relying on self-reported data.

Throughout, we have implicitly assumed that coaching and decision analysis are effective approaches for making career-related decisions. Existing studies measuring the effectiveness of coaching and decision analysis focus mostly on the organizational setting. A systematic review of the research on executive coaching has suggested that it leads to various positive outcomes for the coachee, such as increased self-efficacy, goal attainment, resilience, well-being and reduced levels of stress [42]. Decision analysis, in turn, has been shown to help organizations achieve higher financial performance, create new opportunities, and deal better with uncertainty [43,44]. To date, however, no empirical studies have examined or compared the effectiveness of academic coaching and decision analysis applied to career choice. Future studies comparing the effectiveness of the two approaches could measure the quality of the decision process used as well as the quality of the short- and long-term consequences for the coachee.

Ultimately, an academic’s preference for one approach over the other will be mostly determined by how he or she values the features of each approach. One approach, academic coaching, is a paid service which is assumed to do more for the well-being and performance of academics than just providing them with a systematic decision-making process. The second approach, decision analysis, requires a nonmonetary investment, as time and effort must be put into learning the necessary concepts and techniques before applying them to the career choice problem. Both approaches can, in principle, be applied to other career choice problems, such as deciding whether to do a PhD or deciding between alternative job offers. Although neither approach can be guaranteed to lead to a successful or fulfilling career, their systematic methods can help academics lessen the chances of unpleasant consequences.

## Supporting information

S1 Appendix English and German versions of the interview script. (DOCX)
