# Supplementary material for: Academic coaching and decision analysis: Ways of deciding whether to pursue an academic career
Source: PLoS One. 2018 Nov 15;13(11):e0206961. doi: 10.1371/journal.pone.0206961 (PMC6237325; doi:10.1371/journal.pone.0206961)
Supplement: S1 Appendix — (DOCX) [file pone.0206961.s001.docx]

**S1 Appendix: English and German Versions of the Interview Script**

**Interview Script (English Version)**

*Our project investigates three related questions: 1) how junior academics make career-related decisions, 2) what kind of career concerns they experience during the career planning process, and 3) what academic coaches can do to help. Your answers will be tape recorded for the purpose of scientific analysis. When transcribing your answers, your name and any other information that could allow your identification will be replaced by a code label. Your data will be used solely by the DZHW, in the context of scientific publications.*

***Part I: What academic coaches do***

1. What can an academic coach do for academics that can be distinguished from the mentoring by a senior faculty member?
2. How does a typical coaching consultation look like?

***Part II: Your clients***

1. Who are your clients?
2. Are your clients a representative group of all junior academics in Germany or are they in some way a “special” group of people?
3. Do you follow up on your clients’ career moves after the coaching?

***Part III: The concerns your clients have***

1. What concerns do academics have that make them come to you?
2. In the rest of this interview, I would like us to focus on the career-related concerns that academics have. Do your clients have any other concerns regarding their careers?
3. How do PhD students, postdocs, and junior professors differ in terms of their career concerns?
4. Does people’s discipline of study have an influence on what career concerns they have?

***Part IV: The career coaching services you provide***

1. What information about the client do you usually take into account when preparing the coaching?
2. How do you help your clients find out what kind of career path could suit them?
3. Is your coaching approach any different when clients have a clear vision of what career they want to pursue?
4. Do you sometimes help your clients when they are faced a tough decision and are at a loss as to what is best to do?
5. What do you do if you believe that the career plan of a client is inadequate or unrealistic?

***Part V: Conclusion* (optional)**

1. Would you say that junior academics working in Germany can easily get specialized career-related support, even when they cannot afford the services of an academic coach?
2. How do you perceive the academic and nonacademic job markets in Germany?
3. Why did you become an academic coach?

**Interviewskript (Deutsche Version)**

*Unser Projekt untersucht drei verwandte Fragestellungen: 1) Wie machen junge Akademiker karriere-relevante Entscheidungen? 2) Welche Arten von Karriereproblemen erleben diese während des Prozesses der Planung ihrer Karriere? 3) Was können Wissenschaftscoaches machen, um zu helfen? Ihre Antworten werden auf Band aufgenommen und zum Zwecke wissenschaftlicher Analysen genutzt. Beim Transkribieren ihrer Antworten wird ihr Name und jegliche anderweitige Information, die zu Ihrer Identifikation genutzt werden könnte, anonymisiert. Ihre Daten werden nur durch das DZHW, im Kontext wissenschaftlicher Arbeiten, genutzt.*

***Teil I: Was machen Wissenschaftscoaches?***

1. Was kann ein Wissenschaftscoach für Akademiker tun, was sich von der Betreuung durch ein fortgeschrittenes Fakultätsmitglied unterscheidet?
2. Wie sieht eine typische Coaching-Beratung aus?

***Teil II: Ihre Klienten***

1. Wer sind Ihre Klienten?
2. Sind ihre Klienten eine repräsentative Gruppe aller jungen Akademiker in Deutschland oder sind sie in irgendeiner Weise eine “spezielle” Gruppe von Menschen?
3. Verfolgen Sie die Karriereschritte ihrer Klienten nach dem Coaching?

***Teil III: Die Probleme ihrer Klienten***

1. Aufgrund welcher Probleme kommen Akademiker zu Ihnen?
2. In der restlichen Zeit des Interviews, werden wir uns auf die karriererelevante Probleme von Akademikern fokusieren. Haben Ihre Klienten irgendwelche anderen Probleme in Bezug auf ihre Kariere?
3. Wie unterscheiden sich Doktoranden, Postdoktoranden, und Juniorprofessoren in ihren Karriereproblemen?
4. Hat die Studierichtung einen Einfluss auf die Karriereprobleme einer Person?

***Teil IV: Die Karriere-Coaching-Dienstleistungen, die Sie anbieten***

1. Welche Informationen über den Klienten nutzen Sie üblicher Weise, wenn Sie sich auf ein Coaching vorbereiten?
2. Wie helfen Sie ihren Klienten den Karrierepfad zu finden, der zu Ihnen passen könnte?
3. Ist ihr Coachingansatz anders, wenn ihre Klienten eine klare Vorstellung von der Karriere haben, die sie verfolgen wollen?
4. Helfen Sie manchmal ihren Klienten, wenn Sie vor einer schweren Entscheidung stehen und nicht wissen, was sie am besten tun sollten?
5. Was tun Sie, wenn Sie glauben, dass der Karriereplan eines Klienten inadequat oder unrealistisch ist?

***Teil V: Fazit* (optional)**

1. Würden Sie sagen, dass junge Akademiker, die in Deutschland arbeiten, leicht spezialisierte karriererelevante Hilfe finden können, auch wenn Sie sich die Dienstleistungen eines Wissenschaftscoaches nicht leisten können?
2. Wie nehmen Sie den akademischen und nicht-akademischen Arbeitsmarkt in Deutschland wahr?
3. Warum sind Sie Wissenschaftscoach geworden?
4. Warum sind Sie Karrierecoach geworden?
